# Case report: Exploring the utility of whole-body bone scintigraphy for pediatric Langerhans cell histiocytosis: insights from clinical practice

**DOI:** 10.3389/fonc.2024.1294772

**Published:** 2024-02-09

**Authors:** Wenyu Song, Fan Hu, Wei Shi, Fang Wang, Yongxue Zhang, Xiaoli Lan, Xiaotian Xia

**Affiliations:** ^1^ Department of Nuclear Medicine, Union Hospital, Tongji Medical College, Huazhong University of Science and Technology, Wuhan, China; ^2^ Hubei Province Key Laboratory of Molecular Imaging, Wuhan, China; ^3^ Institute of Hematology, Union Hospital, Tongji Medical College, Huazhong University of Science and Technology, Wuhan, China; ^4^ Department of Nuclear Medicine, Wuhan Children’s Hospital (Wuhan Maternal and Child Healthcare Hospital), Tongji Medical College, Huazhong University of Science and Technology, Wuhan, China

**Keywords:** Langerhans cell histiocytosis, pediatric, ^99m^Tc-MDP, bone scan, skeletal LCH

## Abstract

**Purpose:**

This mini-review delves into the realm of Langerhans cell histiocytosis (LCH) in children, focusing on its skeletal involvement. By synthesizing pertinent literature, we sought to provide a comprehensive understanding of LCH’s clinical and radiographic spectrum. Our study then demonstrates the diagnostic prowess of whole-body ^99m^Tc-methyl diphosphonate (MDP) scintigraphy in LCH cases, underscoring its value in tandem with existing knowledge.

**Methods:**

Our approach involved an extensive literature review that contextualized LCH within the current medical landscape. Subsequently, we presented a case series featuring five pediatric instances of skeletal LCH, one accompanied by soft tissue infiltration. The principal aim was to illuminate the diagnostic and staging potential of whole-body ^99m^Tc-MDP scintigraphy, augmenting existing insights.

**Results:**

Through meticulous literature synthesis, we highlighted pediatric LCH’s protean clinical manifestations and radiological variability. Aligning with this spectrum, our case series underscored the role of ^99m^Tc-MDP scintigraphy in diagnosing and staging LCH. Among the five pediatric cases, one demonstrated concurrent soft tissue involvement. This aligns with the multifaceted nature of LCH presentations.

**Conclusion:**

Pediatric LCH can present with a wide range of clinical and radiologic features. By amalgamating our cases with extant literature, we stress the necessity of a multimodal strategy. ^99m^Tc-MDP scintigraphy emerged as an indispensable tool for accurate staging and soft tissue detection. Our findings collectively advocate for a holistic approach to managing LCH, ensuring informed therapeutic decisions for optimal patient outcomes.

## Introduction

Langerhans cell histiocytosis (LCH) is a rare disorder that results from abnormal clonal proliferation of macrophages. These cells infiltrate various tissues such as the bone marrow, skin, central nervous system, lung, liver, spleen, and lymph nodes. Multi- and unifocal forms of the disease have been described (1). Among pediatric patients, the site most affected is the skull, followed by the spine, extremities, pelvis, and ribs (2). Clinical symptoms of bone lesions include pain, swelling, and pathological fracture (3).

LCH lesions typically appear sharp-edged and osteolytic on plain radiography, which is the primary means of diagnosis and disease staging. Computed tomography (CT) and/or magnetic resonance imaging (MRI) can also be used for diagnosis, delineation of bone destruction, and showing marrow and soft tissue involvement (4). ^99m^Tc-methyl diphosphonate (MDP) scintigraphy can be used to show the distribution of radioactivity in diseased bones, which can help determine disease stage (3).

We retrospectively analyzed whole-body ^99m^Tc-MDP bone scan data in five children with LCH to show the value of systemic bone imaging in LCH diagnosis and staging. Our findings underscore the importance of utilizing a multimodal approach in pediatric LCH patients.

## Case presentations

### Case 1

**Figure 1:**
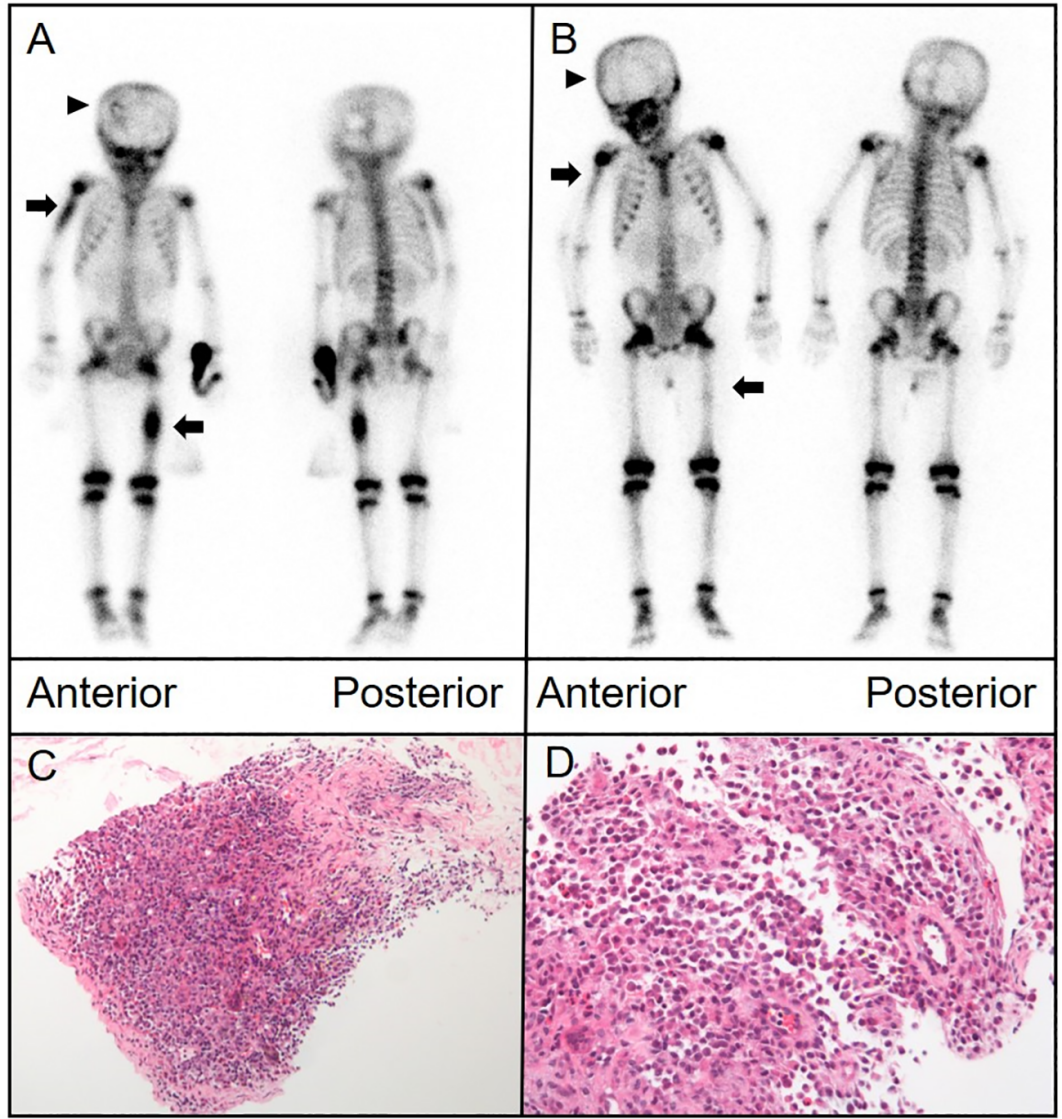
**(A)** Anterior and posterior bone scintigraphy images revealed marked uptake of the radiotracer in the right humerus and left femur, indicated by arrows, alongside an area of reduced uptake in the left skull, marked by an arrowhead. **(B)** Follow-up images taken 18 months later exhibited noticeable changes in the radiotracer uptake within these lesions. **(C, D)** Histological analysis of the proximal humerus section, stained with hematoxylin and eosin, displayed a proliferation of histiocytes with folded nuclei and a scarcity of mitotic figures. [**(C)**,×40; **(D)**,×100].

A 5-year-old girl presented with a hard, purplish red mass on her right shoulder that was tender to palpation. Her condition temporarily improved for only 1 month after antibiotic administration. Biopsy suggested LCH. A ^99m^Tc-MDP bone scan demonstrated uptake abnormalities in the skull, right humerus, left femur, thoracic vertebrae, right ribs, lumbar vertebrae, and right sacroiliac joint. Bone density was low in the lesions on CT. A follow-up bone scan 18 months later revealed new metabolically active lesions; however, some pre-existing lesions had improved slightly (**Figure 1**).

### Case 2

**Figure 2:**
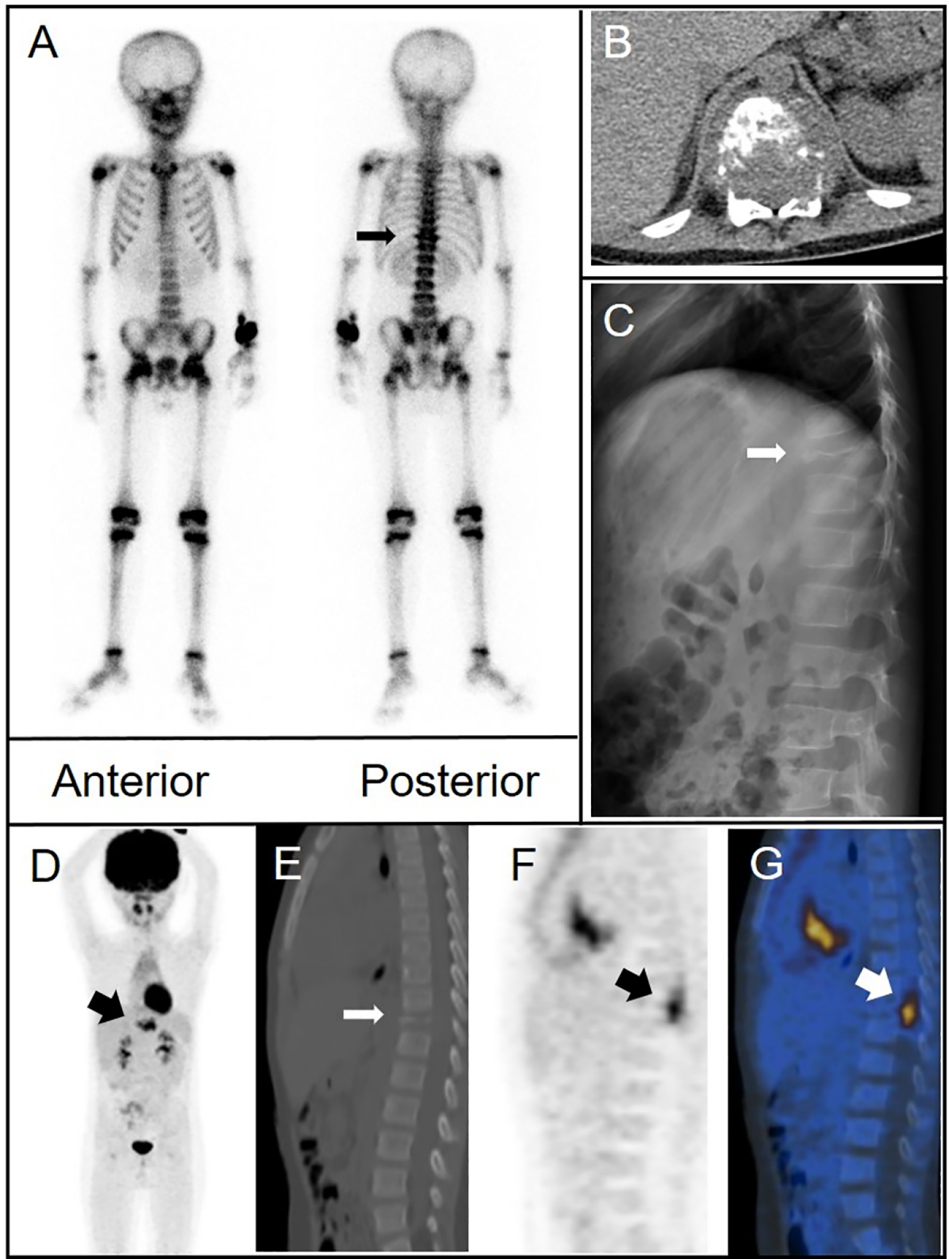
**(A)** Bone scintigraphy (anterior and posterior) demonstrated avid radiotracer uptake in the T11 vertebral body (arrow). **(B)** Axial CT imaging identified bone destruction at T11 with partial cortical breach and an adjoining paravertebral soft tissue mass that contributed to spinal canal stenosis. **(C)** Lateral radiograph of the spine demonstrated osteolytic damage and collapse of the T11 vertebral body (arrow). **(D–G)** ^18^F-fluorodeoxyglucose (FDG) positron emission tomography (PET)/CT showed extensive destruction of the T11 vertebral body (thin arrow) and intense FDG uptake (maximum standardized uptake value of 8.8) in the surrounding soft tissue (thick arrows) [**(D)**, MIP image; **(E)**, CT; **(F)**, PET; and **(G)**, fusion].

A 6-year-old boy with progressively worsening paroxysmal lumbago was referred to our hospital for treatment. His pain was exacerbated by activity. Physical therapy had not provided any significant relief. He also reported soreness in both feet and thighs and a burning sensation in the soles of his feet. Plain radiography of the spine revealed an osteolytic lesion with cortical breakthrough and vertebral collapse in the T11 vertebral body. MRI of the spine indicated soft tissue invasion of the bone. A ^99m^Tc-MDP bone scan revealed mild uptake in the T11 vertebra. CT showed a flattened T11 vertebral body with cortical perforation and uneven coin-like changes in bone density. Signal density in the soft tissues surrounding the vertebra appeared slightly higher in a patchy pattern. ^18^F-fluorodeoxyglucose PET/CT showed bone destruction and abnormal metabolism in the T11 vertebral body and thickening of the surrounding soft tissue. Pathological examination of a biopsy specimen confirmed LCH (**Figure 2**).

### Case 3

**Figure 3:**
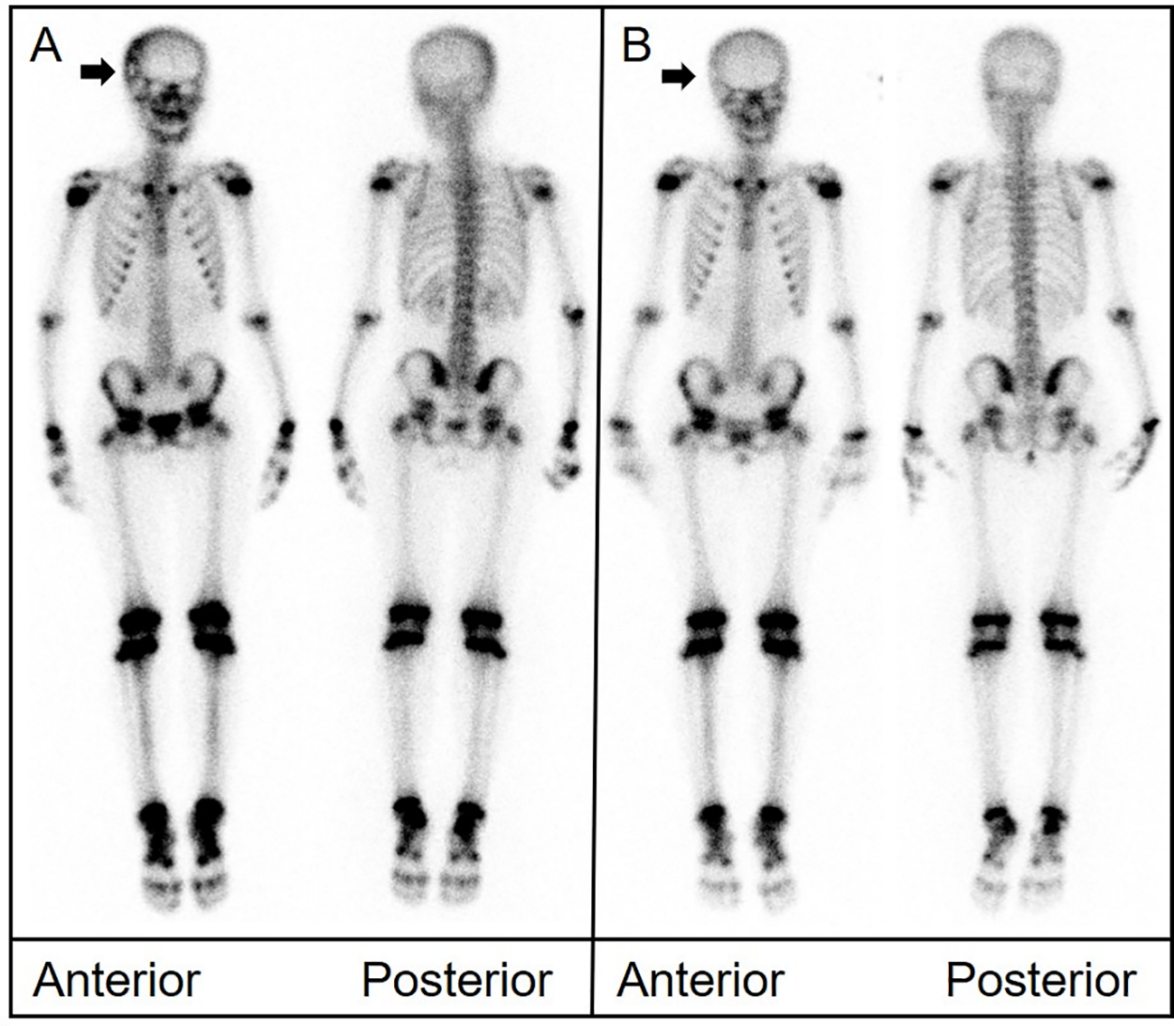
**(A)** Bone scintigraphy (anterior and posterior) displayed a region in the right temporal bone with high peripheral and low central radiotracer uptake (arrow). **(B)** Imaging conducted 1 year later revealed a more even distribution of radioactivity in the same area.

A 10-year-old girl presented with a tough and tender mass in the right temporal region. CT showed bone destruction in the area with uneven local density as well as high-density and lamellar low-density shadows in the skull. During surgical excision of the mass, the lesion was noted to extend from the aponeurosis to the dura mater. The temporal bone defect was 3 cm × 2 cm in dimension. The pathological diagnosis was LCH. Postoperative bone scintigraphy revealed sparse central distribution of tracer in the right temporal bone with peripheral accumulation (**Figure 3**). Follow-up imaging 1 year later showed a lower and more uniform concentration of radioactivity in the corresponding area.

### Case 4

**Figure 4:**
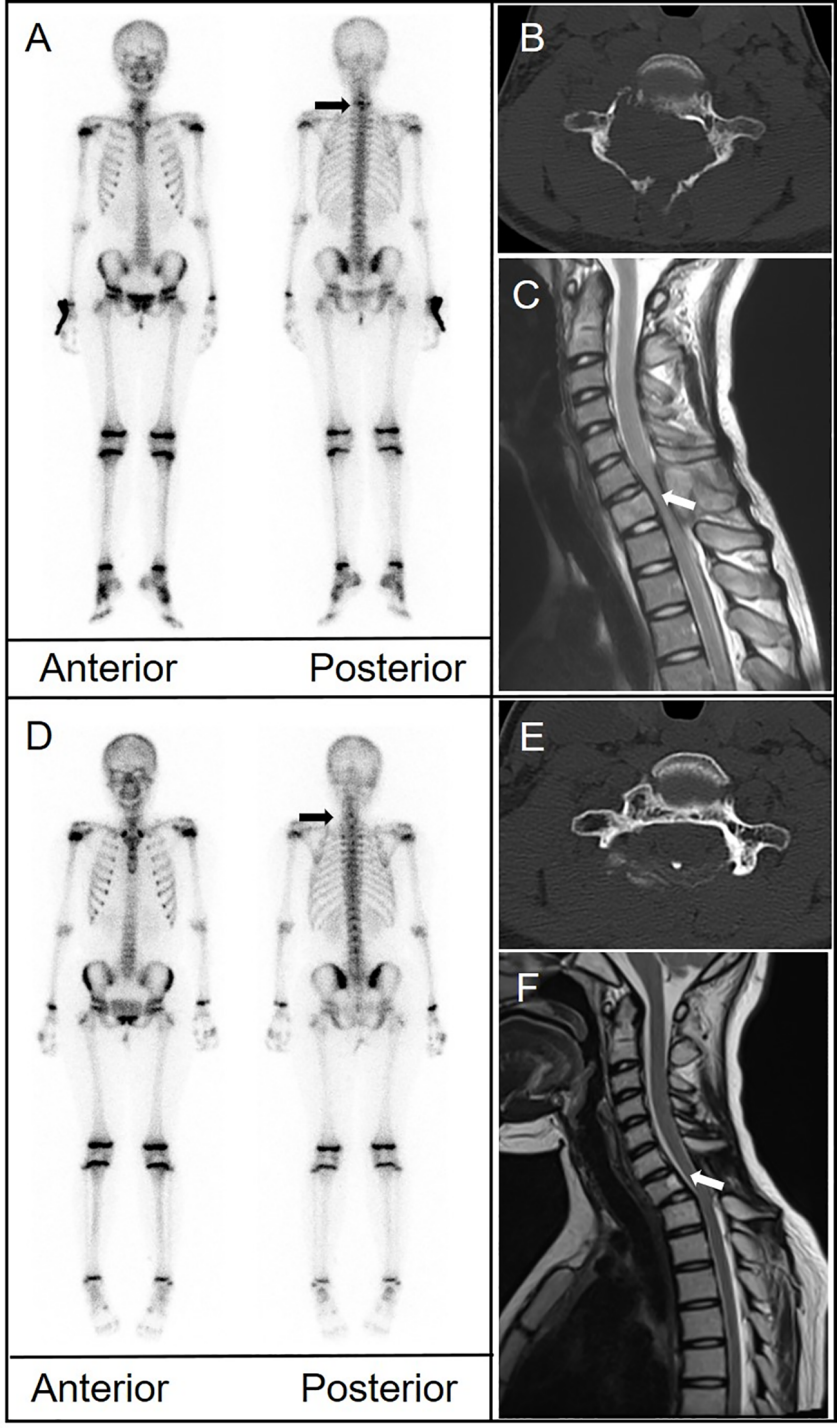
**(A)** Bone scintigraphy (anterior and posterior) demonstrated avid radiotracer uptake in the lower cervical spine (arrow). **(B)** CT depicted significant structural damage to the C7 vertebral body, right pedicle, and posterior elements. **(C)** Magnetic resonance imaging (MRI) showed signal change in the C7 body and posterior elements with associated spinal canal stenosis and cord compression (arrow). **(D)** A follow-up bone scan 11 months later showed disappearance of uptake in the cervical spine. **(E)** CT showed persistent bone destruction. **(F)** MRI showed improvement in spinal canal stenosis.

An 11-year-old girl presented with neck and shoulder pain that worsened during exercise. CT of the cervical spine suggested a space-occupying lesion in the C7 vertebral body and right pedicle. MRI showed signal change in the C7 vertebral body and posterior elements with associated spinal canal stenosis and cord compression. Biopsy was performed. Pathological examination revealed that the tumor cells were positive for CD1a and langerin. The ^99m^Tc-MDP bone scan showed abnormal uptake in the lower cervical spine. Another bone scan 11 months later revealed decreased uptake at C7. CT and MRI showed persistent osteolytic destruction but improvement in spinal canal stenosis (**Figure 4**).

### Case 5

A 9-year-old boy with limited neck movement and pain despite treatment was referred for further evaluation. Biopsy of a cervical tumor revealed cells positive for CD1a, s-100, and langerin. A ^99m^Tc-MDP bone scan showed mildly increased uptake in the middle of the cervical spine. CT and MRI suggested a C3 paraspinal soft tissue mass with associated bone destruction. Two years after surgery, follow-up scintigraphy and MRI were normal.

## Discussion

LCH tends to present in children with a broad spectrum of clinical and radiologic features. The reported incidence of LCH in children under 15 years of age is from 2.6 to 8.9 cases per million per year; most children are between 0 and 3 years of age at the time of diagnosis (5). Mean age of the patients in our series was 8 years.

The skeleton is affected in 75% to 80% of children with LCH (6). More than half of all bone lesions occur in the flat bones, cranium, pelvis, or ribs. The skull is the most involved site. Skull lesions are typically round or ovoid, “perforated” in appearance, and have well-defined non-sclerotic margins (7). Long bone lesions have a medullary destruction zone in the early stage, then gradually expand to erode the intimal cortex in a “scallop shape”. In the spine, LCH lesions are lytic in the early stage, which causes vertebral body collapse and widening of the intervertebral space. With healing, the involved vertebra is reconstituted toward the original height (8). The presence of both osteolytic and osteogenic bone destruction on imaging may indicate LCH. In LCH involving the spine, ^99m^Tc-MDP scintigraphy is characterized by significant tracer uptake in the affected spinal segments. Planar bone imaging of the skull is associated with a certain false-negative rate because of the skull’s structure. Therefore, it should be combined with other imaging modalities and/or lesion biopsy to improve diagnostic accuracy. In our series, ^99m^Tc-MDP scintigraphy was positive in all five patients and a total of 10 scans were performed. Three of the lesions involved the skull and mainly manifested as a circular zone of increased uptake. Abnormal uptake in the spine was observed in 12 locations (cervical vertebrae, 2; thoracic vertebrae, 7; and lumbar vertebrae, 3). In the three long bone lesions, all were in the diaphysis and presented as a “strip-fusiform” zone of abnormal uptake. In the single rib lesion, a “strip-fusiform” uptake zone was observed owing to bone dilatation.

LCH is classified as single-systemic or multisystemic (**Table 1**). Treatment options and prognosis differ between the types. Single bone lesions are more common in single-system (SS) LCH and generally have a good prognosis. Distinguishing SS LCH from multisystem (MS) LCH is important (9). Previously, LCH bone lesions were classified into three categories: group I, solitary bone lesion; group II, multiple bone lesions without systemic involvement; and group III, multiple bone lesions with systemic involvement. SS LCH and MS LCH are equivalent to groups II and III, respectively. Whenever bone LCH is diagnosed, it is important to determine the number of bone lesions, number of systems involved, and whether there is involvement of “risk” bones such as the temporal, ear-petrous, facial, and orbital bones as well as any other skull or facial lesions (2). These factors must also be re-evaluated during follow-up. ^99m^Tc-MDP scintigraphy can evaluate for lesions throughout the entire body and has shown its value in preliminary staging. In our series, the imaging characteristics of solitary LCH lesions on ^99m^Tc-MDP scintigraphy were mainly characterized by local uptake, which varied according to location. For example, LCH lesions in the skull were annular foci. Multiple lesions were seen in the skull, spine, and long bones, often with surrounding soft tissue invasion. All five patients had SS LCH (group II) involving only the skeletal system. Four patients had a single skeletal lesion that disappeared after treatment. The remaining patient had multiple skeletal lesions in the skull, long bones, spine, ribs, and pelvis and new lesions appeared over 18 months of follow-up. As reported in previous studies, patients with multifocal involvement appear to have a worse prognosis.

**Table 1:** Clinical classification of Langerhans cell histiocytosis.

Type	Single-system LCH (SS-LCH)	Multisystem LCH (MS-LCH)
Organ or site	**Bones** (single bone, single or multiple, and many foci in many bones)	**Involvement of critical organs** (the hemopoietic system, spleen, liver, and the central nervous system)
	**Skin**	**No involvement of critical organs**
	**Lymph node** (excluding the lymph node draining the area of histiocyte infiltrate) or multiple lymph nodes (more than one lymph node group)	
	**Hypothalamus**	
	**Isolated pulmonary involvement**	
	**Other** (thyroid and thymus involvement)	

Reported sensitivities of bone scintigraphy for LCH diagnosis range between 35% and 60% (10). Such high false-negative rates may be related to the fact that lesions in the pelvis and skull and those near a growth plate are difficult to detect (11). Additionally, lesion size and characterization can also impact sensitivity. For example, lesions with a purely osteolytic aspect without radiographic evidence of osteoblastic reaction may be missed by bone scintigraphy. CT or MRI is recommended if LCH is suspected and scintigraphy is negative. Bone scintigraphy is less useful for primary detection and diagnosis of LCH; its main role is in evaluating treatment response and detecting recurrence. In our study, ^99m^Tc-MDP uptake changed considerably during follow-up in three patients, and even returned to normal in one. Osteolysis and osteogenesis can exist at the same time in LCH and follow-up is necessary for at least 3 years or until resolution of symptoms or the lesion itself (12). As shown in our study and in others, changes in bone scintigraphy reflect changes in LCH bone lesions, and these changes have treatment implications. Whole-body ^99m^Tc-MDP bone scintigraphy may be a sensitive method for detecting clinically active lesions.

In the realm of LCH, the prospect of imaging techniques holds particular promise for advancing our understanding and management of the disease. As current research pivots towards precision diagnostics, future studies are likely to expand on the use of sophisticated imaging modalities such as advanced PET tracers that target specific LCH markers. These could offer a more nuanced picture of disease activity and potentially unveil subclinical manifestations. Additionally, integrating these advanced imaging techniques with the burgeoning field of radiomics could yield predictive models that forecast disease progression and response to treatment. The continued exploration of imaging in LCH not only aims to refine diagnostic accuracy but also endeavors to personalize therapeutic regimens, ensuring optimal patient-centric outcomes.

There are several limitations in this study. The sample size is relatively small, with only five pediatric cases, which may not fully represent the spectrum of LCH in the larger population. Additionally, our findings are retrospective and may be subject to selection bias. Future research with a larger cohort and a prospective design would be beneficial to validate and expand upon our results.

## Conclusion

Children with suspected SS LCH involving the skeleton should undergo a comprehensive diagnostic examination to rule out multiple lesions. Although the sensitivity of ^99m^Tc-MDP bone scintigraphy for diagnosis of LCH is low, it has an important role in excluding other diagnostic possibilities and in follow-up, as it can demonstrate disease course, guide treatment, and predict outcome by distinguishing active lesions.

## Data availability statement

The raw data supporting the conclusions of this article will be made available by the authors, without undue reservation.

## Ethics statement

The studies involving humans were approved by Committee of Union Hospital, Tongji Medical College. The studies were conducted in accordance with the local legislation and institutional requirements. Written informed consent for participation in this study was provided by the participants’ legal guardians/next of kin. Written informed consent was obtained from the individual(s) for the publication of any potentially identifiable images or data included in this article.

## Author contributions

WYS: Data curation, Writing – original draft, Writing – review & editing. FH: Data curation, Formal analysis, Methodology, Software, Writing – review & editing. WS: Supervision, Writing – review & editing. FW: Data curation, Resources, Writing – review & editing. YZ: Data curation, Supervision, Writing – review & editing. XL: Funding acquisition, Project administration, Supervision, Writing – review & editing. XX: Investigation, Supervision, Writing – review & editing.
